# Sex Differences in Echocardiographic Characteristics of COVID-19 Patients

**DOI:** 10.1016/j.jacadv.2023.100440

**Published:** 2023-07-27

**Authors:** Brigitte Kazzi, Vivek Jani, Elie W. Akl, Rimsha Rana, Nisha A. Gilotra, Nicole Bavaro, Thomas S. Metkus, Allison G. Hays, Monica Mukherjee

While male sex is a risk factor for COVID-19 disease severity, the relationship between sex and myocardial function in COVID-19 remains unclear.^1^^,^^2^ We investigated sex differences in echocardiographic parameters of ventricular function and hospitalization outcomes in COVID-19 patients.

We reviewed data from 448 patients hospitalized for acute COVID-19, who underwent a clinically indicated transthoracic echocardiogram (TTE) during hospitalization from March 2020 through January 2021. Data and routine clinical laboratory data were collected from the electronic health record. Medicare Social Security index was used for mortality. Informed consent was obtained and required by the Johns Hopkins Institutional Review Board.

TTE examinations (Vivid E9 and Vivid E95 ultrasound systems, GE Medical) were performed in accordance with American Society of Echocardiography guidelines. Left ventricular (LV) volumes were indexed to body size. Right ventricular (RV) dysfunction was defined quantitatively using tricuspid annular plane systolic excursion and RV end-diastolic dimension and qualitatively by degree (normal, mild, moderate, severe).

Our primary end points were LV ejection fraction (LVEF) and RV dysfunction. Secondary end points were in-hospital all-cause mortality, intensive care unit admission, and hospital length of stay. Variables were compared with a Mann-Whitney U test or chi-square test, where appropriate. Multivariable regression body mass index (assumptions checked and verified) was used to evaluate the association of LVEF and RV dysfunction with age, race, days from admission to index TTE, and a linear support vector machine was trained from all echocardiographic features measured. Linear coefficients were extracted as a proxy for variable importance.

The cohort consisted of 233 (52%) females, the majority of whom were Black (52%), with a mean age of 64.2 ± 15.1 years (Table 1). There were no significant differences in cardiovascular comorbidities between sex. After covariate adjustment for age, race, and body mass index, and time from hospitalization to index TTE, LVEF (*P* < 0.0001) and global LV longitudinal systolic strain (LVLSS) (*P* < 0.0001) were depressed in males whereas RV dysfunction was more common in females (22% vs 11%, *P* = 0.002) despite RV systolic pressure and RV end-diastolic dimension being similar. Females were more likely admitted to the intensive care unit (87% vs 80%, *P* = 0.05). The top 4 predictors for male sex from our support vector machine model were LVEF, LV outflow tract Vmax, left atrial diameter, and global longitudinal strain, while those for female sex were RV systolic pressure, RV outflow tract velocity time integral, A-wave velocity, and E/e’.

**Table 1:** Baseline Characteristics, Echocardiographic Findings, and Clinical Outcomes

	Overall (N = 448)	Male (n = 215)	Female (n = 233)	*P* Value∗
Demographics				
Age (y)	62.0 ± 14.2	59.6 ± 15.0	64.2 ± 15.1	**0.0005**
BMI (kg/m^2^)	30.8 ± 8.7	30.8 ± 8.8	30.8 ± 8.6	0.75
Race				**0.001**
White	122 (27%)	50 (23%)	72 (31%)	
Black	222 (50%)	102 (48%)	120 (52%)	
Asian	15 (3%)	4.0 (2%)	11 (5%)	
Other	64 (14%)	40 (19%)	24 (10%)	
Hispanic ethnicity (N = 448)	76 (17%)	52 (24%)	24 (10%)	**<0.001**
Diabetes mellitus	200 (45%)	87 (40%)	113 (49%)	0.15
CAD	378 (84%)	33 (15%)	35 (15%)	0.81
Hypertension	341 (76%)	154 (72%)	187 (80%)	0.070
Obesity	207 (47%)	110 (52%)	98 (43%)	0.896
History of heart failure	48 (20%)	25 (18%)	23 (22%)	0.52
Chronic kidney disease	90 (28%)	45 (25%)	45 (31%)	0.37
End-stage renal disease	20 (10%)	8.0 (7%)	12 (14%)	0.19
COPD	21 (11%)	11 (10%)	10 (12%)	0.62
Smoking	12 (6%)	9.0 (8%)	3.0 (3%)	0.33
Creatinine (mg/dL) (N = 193)	1.95 ± 2.23	2.07 ± 3.02	1.79 ± 2.23	0.12
Troponin I adm (ng/mL) (N = 218)	0.23 ± 1.39	0.11 ± 0.32	0.38 ± 2.03	0.94
Troponin I echo (ng/mL) (N = 348)	0.37 ± 1.66	0.28 ± 1.12	0.45 ± 2.01	0.45
Troponin I peak (ng/mL) (N = 183)	0.72 ± 2.55	0.54 ± 1.55	0.96 ± 3.47	0.76
NT-Pro-BNP (pg/mL) (N = 379)	9,668 ± 25,242	4,639 ± 20,939	9,669 ± 28,424	**0.0018**
CRP adm (mg/dL) (N = 204)	34.15 ± 61.6	39.71 ± 68.58	26.38 ± 49.70	0.49
CRP echo (mg/dL) (N = 352)	14.10 ± 36.19	13.82 ± 27.06	14.35 ± 42.87	0.21
CRP peak (mg/dL) (N = 401)	29.30 ± 59.50	35.00 ± 64.29	23.95 ± 54.19	0.53
IL-6 (pg/mL) (N = 145)	1,174 ± 4,760	830 ± 1,949	1,689 ± 7,146	0.35
Echocardiographic findings				
RVSP (mmHg) (N = 251)	40.1 ± 15.7	40.26 ± 13.71	39.21 ± 17.29	0.34
RVEDD (base, cm) (N = 385)	3.62 ± 0.71	3.65 ± 0.78	3.58 ± 0.62	0.28
PCWP (mmHg) (N = 260)	17.08 ± 17.97	15.46 ± 17.29	20.55 ± 19.11	**0.0080**
LVEF (%) (N = 399)	57.83 ± 12.03	50.87 ± 11.85	66.05 ± 5.03	**<0.0001**
E/e’ (N = 260)	13.70 ± 13.35	13.26 ± 13.89	14.24 ± 12.72	0.20
RV dysfunction (N = 390)				
Normal	326 (84%)	180 (90%)	146 (78%)	**0.002**
Mild	53 (14%)	16 (8%)	37 (20%)	**0.001**
Moderate	3.0 (1%)	1.0 (1%)	2.0 (1%)	0.611
Severe	6.0 (2%)	4.0 (2%)	2.0 (1%)	0.686
GLVLSS (%) (N = 228)	−16.12 ± 4.89	−15.06 ± 4.82	−17.49 ± 4.66	**0.0013**
TR maximum velocity, cm/s (N = 251)	260 ± 54	246 ± 46	271 ± 59	0.1013
PVR, WU (N = 251)	6.2 ± 1.3	6.5 ± 1.3	5.9 ± 1.2	**0.0262**
RVOT VTI (N = 251)	15 ± 4	14 ± 4	16 ± 4	**0.0243**
RAP, mm Hg (N = 251)	6 ± 4	7 ± 5	6 ± 4	0.1331
IVC diameter, cm (N = 251)	1.8 ± 0.4	1.8 ± 0.5	1.7 ± 0.3	0.4933
TAPSE, cm (N = 313)	1.9 ± 0.5	1.9 ± 0.4	1.9 ± 0.4	0.2499
TAPSE/PASP (N = 251)	0.7 ± 2.0	0.8 ± 0.2	0.6 ± 0.2	**0.05**
Clinical outcomes				
Length of stay, d	20.27 ± 20.26	19.94 ± 18.57	20.60 ± 22.52	0.74
Cardiac arrest	23 (5%)	12 (6%)	11 (5%)	0.92
PE	31 (7%)	14 (7%)	17 (7%)	0.74
STEMI	5.0 (2%)	3.0 (2%)	2.0 (2%)	0.89
ARDS	130 (43%)	73 (44%)	57 (41%)	0.60
AKI	119 (50%)	74 (54%)	45 (43%)	0.087
ICU admission	358 (84%)	169 (80%)	189 (87%)	**0.050**
Death	78 (18%)	35 (17%)	43 (19%)	0.58

Values are mean ± SD or n (%).

AKI = acute kidney injury; ARDS = adult respiratory distress syndrome; BMI = body mass index; CAD = coronary artery disease; COPD = chronic obstructive pulmonary disease; CRP = C-reactive protein; ICU = intensive care unit; IL = interleukin; IVC = inferior vena cava; GLVLSS = global left ventricular longitudinal strain; LVEF = left ventricular ejection fraction; NT-pro-BNP = N-terminal-pro hormone brain natriuretic peptide; PASP = pulmonary artery systolic pressure; PCWP = pulmonary capillary wedge pressure; PE = pulmonary embolism; PVR = pulmonary vascular resistance; RAP = right atrial pressure; RV = right ventricular; RVEDD = right ventricular end-diastolic diameter; RVOT VTI = RV outflow tract velocity time integral; RVSP = right ventricular systolic pressure; STEMI = ST-segment elevation myocardial infarction; TAPSE = tricuspid annular plane systolic excursion; TR = tricuspid regurgitation.

∗ *P* value from Wilcoxon rank sum test.

We demonstrate sex differences in ventricular function in patients hospitalized with acute COVID-19 infection. Male patients were more likely to have depressed LVEF and global longitudinal strain that is not attributable to known confounders, including obesity and age. In females, we observed elevations in RV afterload, including elevated RV systolic pressure. Elevated Doppler A wave velocity was also present in females, suggestive of early grade 1 diastolic dysfunction but did not associate with outcomes. Sex differences in ventricular dimensions, diastology, ventricular remodeling with aging and in response to acute viral infection are known to exist between males and females and may contribute to differences in LV function observed in COVID-19.^2^ Female patients, with baseline more basal diastolic dysfunction, may be more prone to develop RV dysfunction in the setting of an acute illness such as COVID-19, though future studies are required to confirm this hypothesis.^3^^,^^4^

The application of advanced machine-learning techniques employed in our study is novel. Rather than using ML for prediction, our study sought to use it to identify nonlinear relationships between covariates within the population. Utilization of machine learning tools on small data sets with significant noise can allow for unbiased identification of relevant clinical features without the need for large cohorts.^5^

Our study limitations include the observational nature and limited generalizability of a single-center retrospective study, despite the consistency in TTE acquisition and interpretation. Furthermore, qualitative assessment of RV dysfunction is difficult to detect may have limited clinical relevance. Second, clinical outcomes might have occurred before echocardiography. Third, our study only shows a trend for established sex in COVID-19. Fourth, only 56% of patients had TR jets that were adequate for calculations and only 51% of patients had calculated strain; RV strain measurements were not acquired. Fourth, ML techniques, while novel, may lead to overfitting of the data. Lastly, our study population consisted of hospitalized patients who underwent clinically indicated TTE, thus limiting generalizability. Our findings highlight sex-based differences in cardiac function in response to COVID-19 infection. Future studies should investigate long-term sex differences in ventricular function after COVID-19.
